# Using XR (Extended Reality) for Behavioral, Clinical, and Learning Sciences Requires Updates in Infrastructure and Funding

**DOI:** 10.1177/23727322231196305

**Published:** 2023-10-26

**Authors:** Dejan Draschkow, Nicola C. Anderson, Erwan David, Nathan Gauge, Alan Kingstone, Levi Kumle, Xavier Laurent, Anna C. Nobre, Sally Shiels, Melissa L.-H. Võ

**Affiliations:** 1 Oxford Centre for Human Brain Activity, Wellcome Centre for Integrative Neuroimaging, 6396 University of Oxford, Oxford, UK; 2 Department of Experimental Psychology, 6396 University of Oxford, Oxford, UK; 3 Department of Psychology, 8166 University of British Columbia, Vancouver, Canada; 4 Department of Psychology, Scene Grammar Lab, 9173 Goethe University Frankfurt, Frankfurt am Main, Germany; 5 OxSTaR Oxford Simulation Teaching and Research, Nuffield Department of Clinical Neurosciences, 6396 University of Oxford, Oxford, UK; 6 Wu Tsai Institute, 5755 Yale University, New Haven, USA; 7 Centre for Teaching and Learning, 6396 University of Oxford, Oxford, UK

**Keywords:** virtual reality, behavioral insights, clinical sciences, learning sciences, policy

## Abstract

Extended reality (XR, including augmented and virtual reality) creates a powerful intersection between information technology and cognitive, clinical, and education sciences. XR technology has long captured the public imagination, and its development is the focus of major technology companies. This article demonstrates the potential of XR to (1) deliver behavioral insights, (2) transform clinical treatments, and (3) improve learning and education. However, without appropriate policy, funding, and infrastructural investment, many research institutions will struggle to keep pace with the advances and opportunities of XR. To realize the full potential of XR for basic and translational research, funding should incentivize (1) appropriate training, (2) open software solutions, and (3) collaborations between complementary academic and industry partners. Bolstering the XR research infrastructure with the right investments and incentives is vital for delivering on the potential for transformative discoveries, innovations, and applications.

## Tweet

To realize the full potential of XR for cognitive, clinical, and education sciences, we advocate for funding that incentivizes (1) appropriate training, (2) open software solutions, and (3) collaborations between complementary academic and industry partners.

## Key Points

- XR delivers behavioral insights, transforms clinical therapies, and improves education.
- XR applications already support scientific discovery and translation despite major ongoing challenges.
- Realizing the full potential of XR requires investing in its scientific infrastructure.
- Funding schemes should incentivize training, open tools, and collaborations between researchers and industrial partners.
- Policy and funding changes have the potential to deliver transformative discoveries, innovations, and applications.

Extended reality (XR) comprises augmented reality (AR) and virtual reality (VR). These technologies enable users to experience enhanced or virtual environments with the use of head-mounted displays (HMD). In AR, the user has a transparent HMD that overlays images and data onto the real world, enhancing the scope of interaction with the world. In VR, users wear an HMD that completely immerses them in a virtual 3D world. Using controllers, users can interact with and manipulate their environment in a way that is comparable to interactions in the real world (e.g., picking up and moving virtual objects).

The following section exemplifies the potential of XR to (1) deepen the study of human cognition and behavior, (2) transform clinical therapies, and (3) improve learning and education. Subsequent sections outline what currently stands in the way of realizing XR's potential. Finally, we provide recommendations for supporting and guiding XR research to achieve its full scientific, clinical, and translational applications.

## XR Transforms Cognitive and Behavioral Research, Clinical Interventions, and Education

Researchers and practitioners often aim to draw conclusions from findings and develop applications that hold true and apply in different settings, situations, and scenarios (Campbell, 1957; Holleman et al., 2020). For example, attention researchers do not want their findings to be specific to the set of images they selected for their screen-based experiment. Instead, they hope to generalize their insights to attention in the real world (Kristjánsson & Draschkow, 2021; Võ et al., 2019). Similarly, clinicians developing an intervention for agoraphobia in the clinic hope it will be effective in the daily situations that usually trigger anxiety episodes (Freeman et al., 2022; North et al., 1996). In teaching and learning settings, training pilots before they actually have to land a plane for the first time should be as realistic and transferable as possible. XR, and particularly VR, has proven to be a powerful tool in all these cases, because of its immersive realism and the many possibilities for response generation and interaction with the environment (Tarr & Warren, 2002). The rich possibilities for interaction make XR ideal for creating and studying behavior in diverse settings, situations, and scenarios (Draschkow, 2022).

## Delivering Cognitive and Behavioral Insights

A small number of behavioral researchers have embraced XR for decades (Hayhoe et al., 2002; Loomis et al., 1999; Pelz et al., 1999; Tarr & Warren, 2002; Van Veen et al., 1998; Warren et al., 2001). Most advantageously, in VR, the researcher has full access to the experimental environment, as well as the participants’ field of view, position in space, and eye, hand, body, and head movements. The rich multivariate behavioral data enable researchers to construct representative settings without sacrificing experimental control (Draschkow, 2022). The full control over the environment and rich knowledge of the participants’ movements, field of view, and locus of attention enables researchers to test hallmark cognitive systems adequately under ecologically valid contexts and demands (Hayhoe, 2017). The various XR behavioral data streams enable relating where someone is looking to where their head is directed in 3D space within the same frame of reference (Bischof et al., 2023; David et al., 2020; Thom et al., 2023). Transporting cognitive and behavioral research from the flat screen to the immersive environment has already led to foundational insights into how people perceive and navigate their environment (Cohen et al., 2020; Doeller et al., 2010; Finnegan et al., 2023; Sato et al., 2006; Warren et al., 2001), how they allocate attention to find things (Helbing et al., 2022; Li et al., 2016), and how people use memory to complete tasks (Draschkow et al., 2021, 2022; Helbing et al., 2020; Laurent et al., 2016)—just to name a few.

## Transforming Treatments for Mental and Physical Health

The promise of XR applications extends beyond discoveries in basic research. Immersive tools can help individuals manage pain and help promote mental and physical health (Freeman et al., 2017; Maples-Keller et al., 2017; Osumi et al., 2017). For example, XR can support patients with PTSD and schizophrenia, as well as lower anxiety in many disorders (Freeman, 2008; Freeman et al., 2008, 2018; Rothbaum et al., 1995, 2000, 2001). A recent example of VR's transformative clinical potential is the automated VR therapy developed to treat agoraphobia and manage distress in patients with psychosis (Freeman et al., 2022). By simulating convincing experiences in real-world contexts (e.g., café, shop, doctor's surgery) within a safe context, VR enables patients to break their long-lasting and life-debilitating pathological avoidance defense mechanisms. Creating such realistic yet safe contexts in the traditional laboratory, clinic, or the real world would be nearly impossible.

## Improving Learning and Education

Another fundamental and valuable arena for XR application is training and education. As a tool, VR goes beyond simply increasing the engagement of trainees through entertainment. Its strength lies in involving participants in the construction of their knowledge rather than the passive intake of facts (Winn, 1993).

Driving and flight simulators constitute some of the earliest use cases of XR (Aginsky et al., 1997; Van Veen et al., 1998). Since then, virtual reality is showing an increasing presence in education (Rojas-Sánchez et al., 2023), because the high level of immersion and interaction (Martín-Gutiérrez et al., 2017; Radianti et al., 2020) positively impacts knowledge gain (Chavez & Bayona, 2018). For instance, teachers in training learn by being exposed to a wide variety of critical classroom scenarios (Glocker et al., 2023). XR can also be used to address inequalities in educational attainment by increasing accessibility (https://oxr.eng.ox.ac.uk/blog/edtechphase1/) and addressing biases (Roswell et al., 2020).

XR plays an important role in the development of education programs aimed at improving procedural-based skills in medical education (Huber et al., 2017; Moro et al., 2021; Moussa et al., 2022). For example, new programs now teach decision-making skills in the care of a sick child. This area of medicine is usually inaccessible to medical students due to the infrequency of presentation and the negative impact that delayed care due to training can cause on a critically unwell child (Gauge, 2021). Understanding the future of virtual reality in all education sectors will require robust research comparing it to established education methods (Shiels et al., 2019) as well as systematically evaluating its validity as an educational tool (Cook & Ellaway, 2015).

## The Mass Adoption of XR

Finally, and critically interlinked with discovery research, clinical work, and education, research-grade consumer systems are on a path to mass adoption (34 million units are projected to be installed in 2024). The increasing affordability and quality of XR systems will make them common in people's homes and more accessible to researchers around the world (e.g., VR is the U.K.'s fastest-growing entertainment and media sector). Cognitive researchers will thus have the unique opportunity to diversify their samples (Aarts et al., 2015; Chandler & Paolacci, 2017; Henrich et al., 2010) and make interventions, training, and education applications broadly accessible. With its mass appeal, XR ensures a much wider more representative population that can participate in and benefit from cognitive, clinical, and education sciences (Draschkow, 2022).

## Challenges to Realizing the Full Potential of XR

The positive examples of how XR has supported discovery sciences and applied innovations have been achieved despite major ongoing challenges. The most pressing challenge, and the focus of this article, is the lack of appropriate resources and expertise. Particularly, when it comes to implementing software solutions, researchers and practitioners are faced with a persistent shortage of technological know-how. Further, most research groups and practitioners struggle to design realistic immersive content and keep up with new hardware. Consider these challenges in turn.

## Software Challenges

Developing smooth and true-to-life interactions within immersive environments requires a high degree of specialized technological expertise. To meet this requirement, game and entertainment companies have teams of dedicated 3D game developers, animators, and programmers. The reality of most research groups at universities looks different. Here, often a single researcher is expected to fulfill all these roles, while also conducting the research and analyzing the resulting multivariate data. The difficulty and delays of multitasking delay progress and deter many researchers from embracing XR technology. Critically, even in well-established XR laboratories, losing a well-trained researcher due to a job change can have severe ripple effects, as it often takes months/years to train a suitable replacement. This lack of appropriate and dependable expertise is the most substantial challenge to the advancement of XR research and its application.

## Immersive Design Challenges

A related issue is the enormous complexity of designing and creating 3D models and realistic immersive content. Creating virtual worlds and characters requires specialized skills in three-dimensional modeling, texturing, character animation, and programming. While start-ups and companies usually have professionals on staff, most research groups either spend years training personnel or hire external companies to produce virtual content. Either solution is associated with tremendous costs and delays, as the limited budget of most research groups makes them low-priority customers of the hired companies. Indeed, many university-based research groups simply cannot afford to purchase 3D models from some companies. Furthermore, purchased 3D content is often proprietary, and researchers cannot openly share the resulting work with the research community, challenging replicability, and reproducibility.

## Hardware Challenges

Keeping in step with new XR hardware developments is a daunting challenge. XR technology is evolving at breakneck speeds, similar to the rapid technological development of mobile phones in the early 2000s. Major technology companies, as well as many new start-ups, are refining XR systems, making them increasingly affordable to consumers and researchers. Keeping up to date with the most recent tools and validating that applications continue to work reliably are critically important. Therefore, dependable funding streams are required for modernizing equipment and performing validation studies.

## Solutions for Realizing XR Research and Application

Without an appropriate policy, funding, and infrastructural updates, many research institutions will fail to overcome the challenges outlined in the previous section. To realize the full potential of XR for basic and translational research requires improvements across three major domains.

## Support and Train People

Interdisciplinary fellowships, grants, and support schemes targeted at increasing the XR expertise of researchers are at the heart of realizing the potential of XR to improve research and application. Funding should support researchers to increase their XR expertise, including software development and 3D design. In tandem, the already qualified XR experts (across software, design, and hardware) should also be supported in joining research groups that would benefit from their expertise. Critically, all person-related support needs to go beyond short-term commitments and allow for sufficient personal and technical development.

## Fund Open and Freely Available Solutions

An important complementary improvement would be directing XR funding to support the development of freely available software solutions and openly available high-quality 3D models. Currently, many research groups hire expensive companies for software development and design needs, limiting skills training opportunities as well as their budget. In many cases, copyright issues prevent the company-based solutions to be shared with the scientific community. Developing a policy to ensure that contracted XR solutions are shared with the scientific community would be a desirable first step. However, stimulating “in-house” development of software and the design of 3D content is equally important. Ideally, research groups would be able to attract designers to join their groups and create 3D content that is openly available to the wider research community. Free and openly accessible repositories of object and character models would counteract the slow pace of XR adoption in research and help solve copyright issues.

## Fund Collaborations Across Research Institutions, Departments, and Industry

Many research institutions possess the competency to improve their XR capabilities, but these are usually scattered across departments and institutes. Sustainable development of XR solutions and applications requires new links among computer science, engineering, psychology, psychiatry, design and arts, sociology, education sciences, and more. New collaborative hubs need to be established at the confluence of these departments. This can include immersive competency centers combining pools of experts as well as specific research groups who work on these intersections long term. Particularly important would be establishing interdisciplinary XR labs that support the programming and design needs of researchers across the different divisions while at the same time providing training opportunities and access to the newest equipment. Interdisciplinary XR labs could support research groups, provide critical training in emerging technologies to students, and support educators in transforming teaching and training into more immersive formats.

Knowledge transfer and cooperation between research institutions and industry in the XR sector also need to be incentivized. Today, technology industries have access to diverse populations across the globe and some of the richest data on human behavior (Box-Steffensmeier et al., 2022). Immersive worlds are being constructed in service of entertainment and communication on a mass scale. A policy needs to be developed to ensure that behavioral, clinical, and learning sciences can benefit from these resources to advance discoveries and support well-being and learning. Further, creating policies that ensure academics access to the rich data sets provides a unique opportunity for educating industry and the public on data stewardship. As an increasing number of sensors are being added to XR systems, people using the system will become identifiable, and the resulting data might eventually be considered personal health data. Industry must work together with academics and policymakers to strike the right balance. Targeted funding initiatives should open the door to partnerships between the XR industry and research institutions, for developing workable policy solutions that both promote advances and safeguard data privacy.

Finally, the collaborative and interdisciplinary nature of the recommendations just listed will require solutions to administrative hurdles associated with XR practices. Interdisciplinary collaborations are never straightforward. Working across academic institutions in different countries can see significant misalignment of ethical-review procedures and data protection policies. Adding industrial partners to the mix compounds the issues, often introducing issues of nondisclosure and intellectual property. It is important to fund and support administrative streamlining efforts to enable researchers to focus on what they do best: fundamental and applied research.

## Policy Implications

Despite major ongoing challenges, there is convincing evidence that XR technology can be effectively used to deliver discoveries, transform healthcare, and improve education.

To realize the full potential of XR, policymakers should advocate, and funding should incentivize (1) appropriate training, (2) open software solutions, and (3) collaborations between complementary academic and industry partners. Promoting self-sustaining XR research expertise and infrastructure through policy and funding changes will turbocharge the pace of discoveries, innovation, and applications to the great benefit of society.
